# Disruption of peroxisome proliferator-activated receptor α in hepatocytes protects against acetaminophen-induced liver injury by activating the IL-6/STAT3 pathway

**DOI:** 10.7150/ijbs.69609

**Published:** 2022-03-06

**Authors:** Zhenzhen Zhang, Tiantian Yao, Nan Zhao, Hui Liu, Hao Cheng, Frank J Gonzalez, Hua Wang, Guiqiang Wang, Aijuan Qu, Yan Wang

**Affiliations:** 1 Department of Infectious Diseases, Peking University First Hospital, Beijing, 100034, China.; 2 Department of Hepatic Oncology, Xiamen Branch, Zhongshan Hospital, Fudan University, Xiamen, 361015, China.; 3 Department of Physiology and Pathophysiology, School of Basic Medical Sciences, Capital Medical University; Key Laboratory of Remodeling-Related Cardiovascular Diseases, Ministry of Education, Beijing 100069, China.; 4 Department of Pathology, Beijing Youan Hospital, Capital Medical University, Beijing 100069, China.; 5 Laboratory of Metabolism, Center for Cancer Research, National Cancer Institute, National Institutes of Health, Bethesda, 20892, USA.; 6 Department of Pharmacy, Anhui Medical University; Department of Oncology, the First Affiliated Hospital, Institute for Liver Diseases of Anhui Medical University, Hefei, 230032, China.

**Keywords:** Acetaminophen (APAP), Hepatotoxicity, Peroxisome proliferator-activated receptor α (PPARα), Oxidative stress, IL-6/STAT3, Liver injury, Liver repair

## Abstract

**Background & Aims:** Peroxisome proliferator-activated receptor α (PPARα) is a ligand-activated transcription factor abundantly expressed in liver. PPARα activator has been previously reported to protect against acetaminophen-induced hepatotoxicity, but fenofibrate, a lipid-lowering drug that activates PPARα, has a common side-effect causing liver injury. Thus, the exact effect of liver PPARα on drug-induced liver injury remains obscure.

**Methods:** Hepatocyte-specific *Ppara* knockout mice and littermate wild-type control mice were intraperitoneally injected with acetaminophen (400 mg/kg body weight). Blood and liver samples were collected at different time points. We measured phase I and II cytochrome P450 enzymes, glutathione, reactive oxygen species, cytokines including *Il6*, and pSTAT3 by reverse transcriptase quantitative PCR, colorimetric, immunohistochemistry analyses and Western blotting.

**Results:** Hepatic expression of PPARα was significantly decreased in DILI patients. Disruption of the *Ppara* gene in hepatocytes significantly reduced acetaminophen-induced liver injury in mice. ROS production rather than the expression levels of phase I and II cytochrome P450 enzymes was reduced in hepatocyte-specific *Ppara* knockout mice compared to control mice after acetaminophen administration. Mechanistically, hepatocyte-specific *Ppara* knockout mice had upregulated activation of the hepatoprotective pathway IL-6/STAT3 compared to wild-type mice, as evidenced by hepatic *Il6* mRNA levels, hepatic protein levels of STAT3 and phosphorylated STAT3 were much higher in hepatocyte-specific *Ppara* knockout mice than in wild-type mice post acetaminophen injection.

**Conclusions:** Hepatocyte-specific disruption of the *Ppara* gene protects against acetaminophen-induced liver injury by reducing oxidative stress and upregulating the hepatoprotective IL-6/STAT3 signaling pathway.

## Introduction

Acetaminophen (APAP) is the most common over the-counter antipyretic and analgesic medicine, but overdose has become the headmost cause of acute liver failure (ALF), with high mortality and economic burden 1, 2. At therapeutic doses, most APAP is metabolized into nontoxic metabolites by UDP-glucuronosyltransferases (UGT) and sulfotransferases (SULT) 3, 4. A small percentage of it is converted by phase I cytochrome P450 (CYP) enzymes to a chemically reactive metabolite (N-acetyl-p-benzoquinone imine, NAPQI), which can be detoxified by directly binding with glutathione (GSH) or by GSH-s-transferases (GST) 4. However, with APAP overdose, GSH is exhausted, resulting in NAPQI accumulation and covalent binding to mitochondrial proteins, ultimately aggravating mitochondrial dysfunction, which is the key mechanism of APAP hepatotoxicity 5, 6. There are two hit theories, the first hit for mitochondrial GSH depletion and ROS production and the second hit for JNK activation and translocation into mitochondria, aggravating ROS generation and mitochondrial membrane permeability transition (MPT), leading to hepatocyte death 5. However, the treatment is limited to N-acetylcysteine (NAC), which works only by restoring the levels of GSH at the early phase 7. Those who progress to acute liver failure often need liver transplantation 8. There is still a lack of effective targeted drugs for this disease.

Precise control of APAP metabolism into toxic metabolites is essential. Nuclear receptors are transcription factors that serve as xenobiotic-activated receptors and help regulate genes involved in drug metabolism and transporters 9. Peroxisome proliferator-activated receptor (PPAR) α is a member of the nuclear receptor superfamily. Activation of PPARα by its ligands (fatty acid/eicosanoid/fibrates) controls fatty acid metabolism and regulates various biological transformation processes in different tissues, especially in the liver 10. Earlier results demonstrated that a PPARα activator (fibrates) could inhibit APAP hepatotoxicity in WT mice 11, 12 but lacked protection against APAP hepatotoxicity in *Ppara^-/-^* mice 13. However, the mechanism of this protection is controversial. Some studies revealed that pretreatment with clofibrate produced an increase in GSH levels, which promoted the exclusion of the toxic metabolite NAPQI 11, but others evidenced that the protection was not due to altered glutathione homeostasis 14. Using liquid chromatography-mass spectrometry (LC-MS) and multivariate data analysis, it was reported that treatment with APAP induced liver injury and suppressed PPARα-regulated pathways, followed by inhibition of fatty acid oxidation and accumulation of acylcarnitines in the serum, thus promoting continuous oxidative stress in the liver 15. A study by Patterson et al suggests that the PPARα target gene uncoupling protein 2 (UCP2) inhibited APAP-induced elevated reactive oxygen species (ROS) generation 16. However, PPARα is enriched in various tissues with abundant fatty acid oxidation, including the liver, heart, brown adipose tissue, skeletal muscles, intestines and kidneys 10. Therefore, it remains unknown whether the lack of protection against APAP-induced liver injury in PPARα-null mice relies on the contribution of PPARα activity in the liver or in other organs. Furthermore, it is reported that liver biochemistry abnormalities develop during fenofibrate therapy in 5-10% of patients. The clinical pattern of fenofibrate associated liver injury has ranged from an acute, self-limited hepatitis to a more persistent and even chronic liver injury 17. So PPARα activity and its effect in the different cell types needs to be investigated.

In the present study, we aimed to investigate the unexplored role of PPARα in hepatocytes in models of APAP-induced liver injury in mice. We generated *Ppara*^ΔHep^ mice and found that disruption of PPARα in hepatocytes protects against APAP-induced liver injury by decreasing APAP-induced oxidative stress and upregulating the IL-6/STAT3 signaling pathway.

## Materials and methods

### Human Liver Histology and Immunohistochemistry

Eleven drug-induced liver injury (DILI) samples from patients who underwent a liver biopsy at Beijing Youan Hospital and three healthy control (HC) samples from patients who underwent hepatic cyst resection at Peking University First Hospital were analyzed. Human liver sections were immunostained for PPARα using an anti-PPARα antibody (Invitrogen, PA1-822A, US). The number of PPARα^+^ cells in the liver sections was counted in 10 randomly selected fields (×400), and the average of the 10 fields was calculated. The study protocol and the use of bio-samples for research were approved by the Ethics Committee of the Peking University First Hospital.

### Mice

Eight- to ten-week-old male C57BL/6 background mice were used in this study. WT mice were purchased from Vital River Laboratory (Beijing, China). Hepatocyte-specific PPARα knockout (*Ppara*^ΔHep^) mice and control floxed (*Ppara*^fl/fl^) mice were constructed as described previously 18. Briefly, mice with a floxed allele of exon 5* Ppara* were built up by using homologous recombination in ES cells and back-crossed with transgenic mice expressing Cre recombinase regulated by the albumin (Alb-Cre) promoters for at least eight generations to create hepatocyte-specific (*Ppara*^ΔHep^) knockout mice. Genomic DNA was extracted from the mouse tail using PCR to confirm the genotype. All animal care and experiments were approved by the Capital Medical University Animal Care and Use Committee. All institutional and national guidelines for the care and use of laboratory animals were followed.

### APAP-induced liver injury

After fasting overnight, all the mice were intraperitoneally injected with 400 mg/kg fresh solutions of APAP (Sigma-Aldrich, St. Louis, MO) dissolving in warmed saline. Mice were sacrificed at different time points after treatment. Liver and blood samples were collected.

### Biochemical Analysis for Serum Samples

Prior to sacrifice, blood was collected from the inner canthus vein with a lancet into heparin-coated tubes. Plasma was collected by centrifugation (8000 rpm, 5 min, 4 °C). Serum levels of alanine transaminase (ALT) and aspartate aminotransferase (AST) were measured using enzyme dynamic methods with the ALT/GPT Reagent Kit and AST/GOT Reagent Kit (Zhongsheng Technologies, Beijing, China), according to the manufacturer's instructions.

### Liver Histology and Immunohistochemistry

Following euthanasia by overdosage, mouse livers were removed, weighed, and dissected into three parts for histological or biological analysis. Formalin-fixed liver samples were embedded in paraffin, sectioned at 4 µm, and subjected to routine hematoxylin and eosin (H&E) staining following standard methods. Immunohistochemical staining for PPARα and CYP2E1 performed using antibodies against 5-bromo-2- deoxyuridine (BD Bioscience, San Jose, CA), according to the manufacturer's protocol. The following primary antibodies were used: anti-PPARα (Abcam, Cambridge, UK) and anti-Cyp2e1 (Millipore, US). The number of PPARα positive cells in the liver sections was counted in 10 random fields (×200), and the average of the 10 fields was calculated. The average area of CYP2E1 in 10 random fields (×200) were analyzed.

### Real-Time Quantitative Polymerase Chain Reaction (qRT-PCR)

Total RNA was extracted from liver tissues using TRIzol reagent (Life Technologies, USA) according to the manufacturer's instructions. Two micrograms of total RNA were reverse-transcribed into cDNA using the GoScript^TM^ Reverse Transcription System (Promega, USA). qRT-PCR was performed in duplicate for each sample using a CFX Connect Real-Time System (Bio-Rad, USA) with SYBR Green Mix (TaKaRa, Japan). The amplification of specificity was confirmed by melting curve profiles. The mRNA level of β-actin was used as an internal standard. Primer sequences were listed in Table 1.

### Measurement of GSH in Liver Tissues

Hepatic levels of glutathione (GSH) were measured in whole-liver homogenates (50-100 mg each of frozen liver tissue) using a glutathione assay kit (Nanjing Jiancheng Bioengineering Institute, China) according to the manufacturer's protocols.

### Measurement of Reactive Oxygen Stress (ROS) in Liver Tissues

Hepatic levels of malondialdehyde (MDA) were analyzed in freshly collected liver tissue lysate using a Lipid Peroxidation MDA Assay Kit (Beyotime, China) according to the manufacturer's protocols. Superoxide in liver tissues was measured by dihydroethidium (DHE) staining after incubation at 37 °C for 30 minutes (Santa Cruz, CA). 4′,6-Diamidino-2-phenylinodole (DAPI; 7 × 10^-6^ mol/L) was added for 5 min to visualize the nuclei and images captured the following day using an Eclipse 55i microscope (Nikon GmbH, Düsseldorf, Germany) and a Fluoro Pro MP 5000 camera (Intas Science Imaging Instruments GmbH, Göttingen, Germany) at 200-fold magnification. Detection wavelength settings were 406-458 nm for DAPI (blue), and 575-700 nm for DHE (red) imaging.

### Western blotting

Tissues were homogenized in lysis buffer (Applygen, Beijing, China), nuclear extraction was done with a nuclear and cytoplasmic protein extraction kit (KeyGENBioTECH, Nanjing, China). The protein concentration was measured using the bicinchoninic acid protein assay kit (Thermo Scientific, Waltham, MA). Western blot analysis was performed with proteins from liver tissues (60 μg) using anti-STAT3α (1:1000 dilution), phosphorylated STAT3 (1:1000 dilution) (Cell Signaling, Beverly, MA), and PPARα antibodies (Abcam, Cambridge, UK). After incubating with horseradish peroxidase conjugated secondary antibody, the immunocomplexes were visualized with FluorChem-R (ProteinSimple, San Jose, CA). Total protein levels were normalized to β-actin (ACTB), and nuclear protein levels were normalized to Lamin B1.

### Statistical analysis

Data were expressed as means ± SEM. Student's t test was performed to compare two groups in the experiment. The four groups, *Pparα*^fl/fl^ + Saline, *Pparα*^fl/fl^+APAP, *Pparα*^ΔHep^+Saline, and *Pparα*^ΔHep^+APAP, were analyzed by one-way analysis of variance (ANOVA), followed by Tukey's post-hoc test. All data were analyzed using GraphPad Prism 7 software for Windows (La Jolla, CA). *P* values less than 0.05 were considered significant.

## Results

### Decreased PPARα expression in DILI patients than HC

To test the expression level of PPARα in DILI patients and HCs, human liver sections were immunostained for PPARα. As indicated in Supplementary Fig. 1, the mean percentage of PPARα^+^ cells in DILI patients and HCs was 45.1% and 59.2% (P=0.04), respectively, and the expression of PPARα in liver sections was significantly decreased in DILI patients compared with HCs (Supplementary Fig. 1A, B). Consistent with the PPARα expression level, histological analysis showed typical decreased PPARα expression in DILI patients compared with HCs (Supplementary Fig. 1A). The clinical characteristics of the patients and healthy controls were showed in Supplementary Table 1. This finding suggests that PPARα signaling pathway plays a partial role in modulating drug induced liver injury.

### Hepatic *Ppara* is downregulated in WT mice after APAP treatment

**Figure 1:**
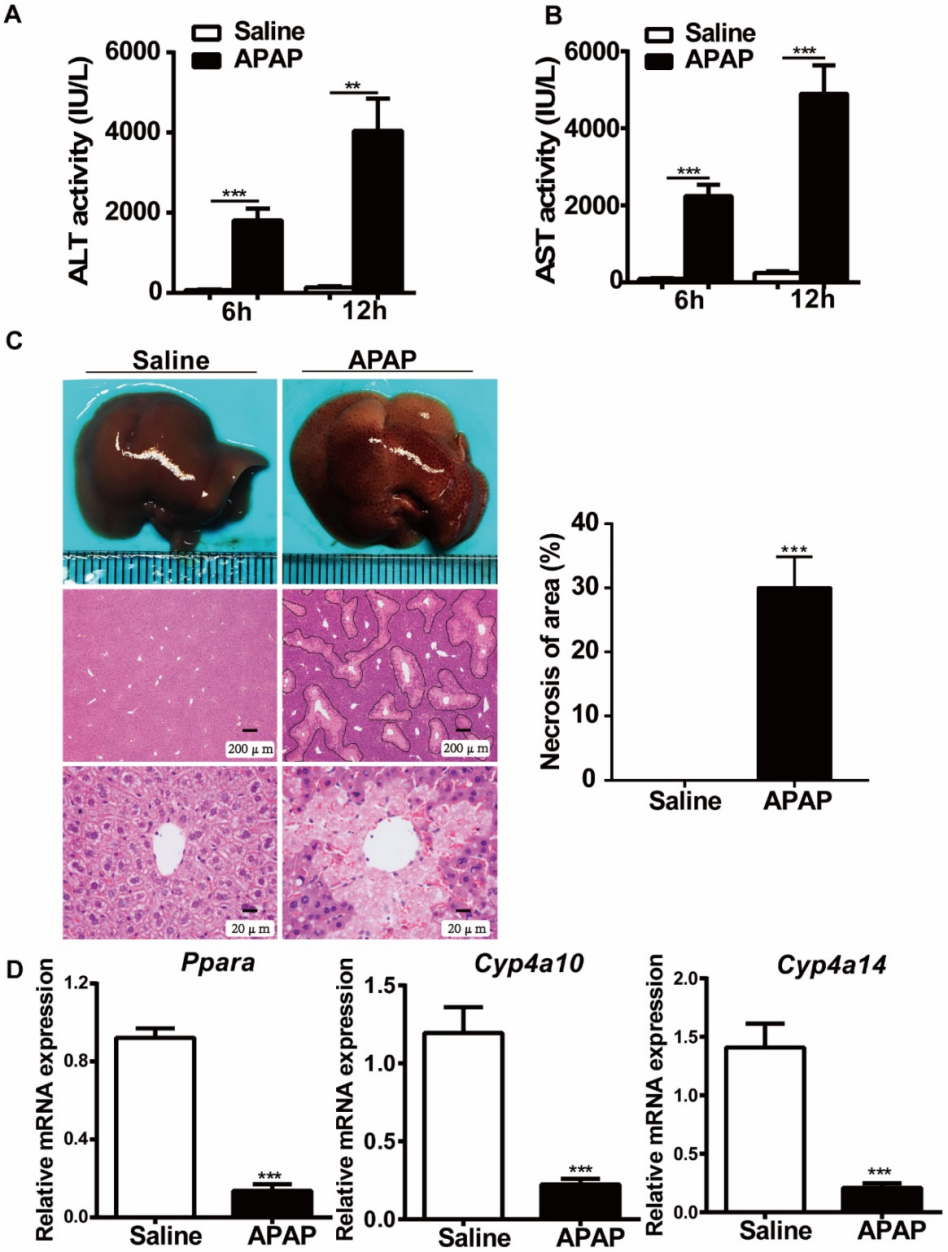
** Hepatic *Ppara* is downregulated in WT mice after APAP treatment.** After overnight fasting, eight-week-old male C57BL/6J WT mice were subjected to intraperitoneal injection with 400 mg/kg APAP or saline. Serum samples were collected at 6 h and 12 h after APAP treatment, and liver tissues were harvested at 12 h after APAP treatment. **(A)** Serum levels of ALT and AST in saline- or APAP-treated mice. **(B)** Representative images of gross livers and H&E staining of saline- or APAP-treated mice. **(C)** Average necrotic areas in livers from saline- or APAP-treated mice. Five fields were randomly selected for analysis. **(D)** qRT-PCR analyses of *Ppara* and its target genes, *Cyp4a10* and *Cyp4a14,* are shown. Data are presented as the means ± SEM (n=5/group), **P<0.01, ***P<0.001.

To examine the effects of APAP treatment on PPARα expression, we first studied the expression of PPARα after administration of toxic doses of APAP (400 mg/kg body weight) to WT mice. As indicated in Fig. 1, the serum levels of ALT and AST were significantly elevated at 6 hours and 12 hours after APAP treatment (Fig. 1A, B). Consistent with the ALT and AST levels, histological analysis established typical necrosis of the area located around the central hepatic veins at 12 hours after APAP treatment (Fig. 1C). Additionally, the relative mRNA expression of *Ppara* as well as *Ppara-*target genes (*Cyp4a10*, *Cyp4a14*) was downregulated in WT mice after treatment with toxic doses of APAP (Fig. 1D), which was consistent with previous studies suggesting that the mRNA expression levels of *Ppara* were inhibited after APAP treatment in WT mice 15, 16.

### Disruption of PPARα in hepatocytes significantly reduces APAP-induced liver injury

**Figure 2:**
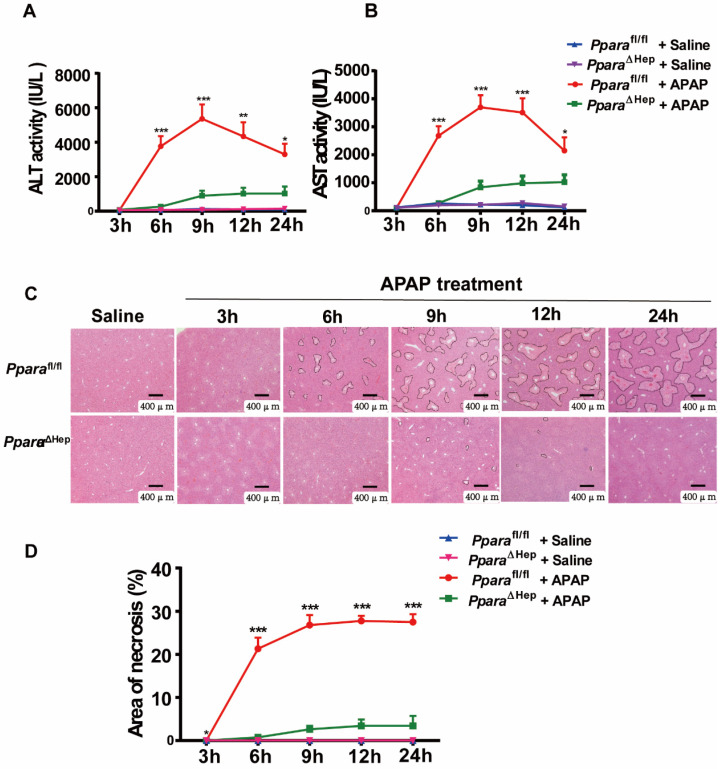
** Disruption of *Ppara* in hepatocytes significantly reduces APAP-induced liver injury.** Eight- to ten-week-old male *Ppara*^ΔHep^ mice and* Ppara*^fl/fl^ mice were subjected to intraperitoneal injection with 400 mg/kg APAP dissolved in warm saline or an equal volume of saline. Liver tissues and serum samples were collected at different time points after APAP treatment. **(A, B)** Serum levels of ALT (A) and AST (B) were measured at different timepoints after APAP challenge. **(C, D)** Representative images of H&E staining (C) and the area of necrosis (D) were examined at various timepoints after APAP challenge. Data were represented as means ± SEM (n=5-10/group). *P<0.05, **P<0.01, ***P<0.001.

**Figure 3:**
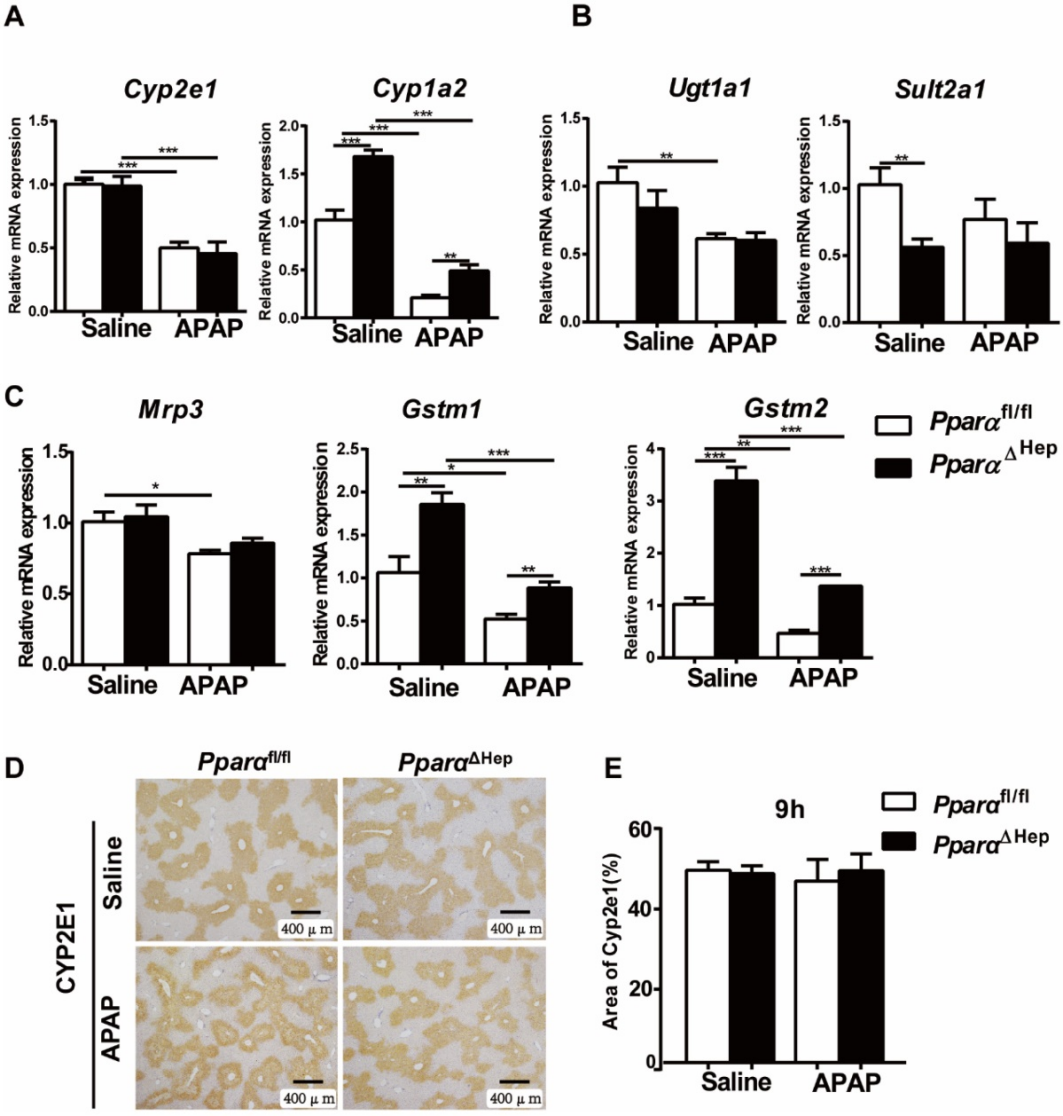
** Disruption of *Ppara* in hepatocytes does not affect APAP metabolism or detoxification.** Eight- to ten-week-old male *Ppara*^ΔHep^ mice and* Ppara*^fl/fl^ mice were treated with APAP or saline. Liver tissues were collected at 9 hours after APAP treatment. qPCR analyses of the expression of phase I metabolizing enzymes (**A**). Phase II metabolizing enzymes (**B**). Drug transporter, GST (phase II metabolizing enzymes) (**C**). IHC of CYP2E1 protein expression (**D**) and the area of CYP2E1 in 5 randomly selected fields (10X) were analyzed (**E**). Data were represented as means ± SEM (n=5-7/group). *P<0.05, **P<0.01, ***P<0.001.

To examine the role of PPARα in hepatocytes in APAP-induced liver injury, *Ppara*^ΔHep^ and littermate control *Ppara*^fl/fl^ mice were administered an overdose of APAP. Hepatocyte-specific PPARα deficiency was confirmed by genotyping, qRT-PCR and Western blot analysis, as previous study. After APAP injection, *Ppara*^fl/fl^ mice showed gradually elevated serum ALT and AST levels as well as extensive liver necrosis, peaking at 9 hours, and then declined progressively but remained at much higher levels at 24 hours after APAP injection (Fig. 2A, B). Interestingly, hepatocyte-specific PPARα deficiency reduced ALT and AST levels at different time points after APAP injection (Fig. 2A, B). In addition, H&E staining revealed that *Ppara*^fl/fl^ mice had a large area of liver necrosis over time, peaking at 9 hours, whereas *Ppara*^ΔHep^ mice strikingly decreased the area of necrosis after APAP injection (Fig. 3C, D). The liver body weight ratio was unchanged in *Ppara*^fl/fl^ and *Ppara*^ΔHep^ mice after APAP injection, as shown in Supplementary Fig. 2. Taken together, these findings suggested that hepatocyte-specific PPAR*α* deficiency significantly reduced APAP-induced liver injury.

### Disruption of PPARα in hepatocytes does not affect hepatic expression of APAP metabolizing enzymes

Previous studies have shown that whether PPARα affects APAP-metabolizing enzymes is controversial 11, 14. To test whether the protection of *Ppara*^ΔHep^ mice against APAP-induced liver injury relied on the alteration of APAP metabolism, the expression of phase I, II, and III hepatic metabolizing enzymes was examined. After APAP injection, the mRNA levels of the metabolic enzymes *Cyp2e1, Ugt1a1, Sult2a1, GSTM1, GSTM2*, drug transporter *Mrp3* as well as the protein levels of CYP2E1, a major P450 in the conversion of APAP into NAPQI, were not significantly different from those in *Ppara*^fl/fl^ mice. Although the expression of the *Cyp1a2*, *Gstm1* and *Gstm2* genes was higher in *Ppara*^ΔHep^ mice than in *Ppara*^fl/fl^ mice, this tendency was also observed under basal conditions without APAP treatment (Fig. 3).

**Figure 4:**
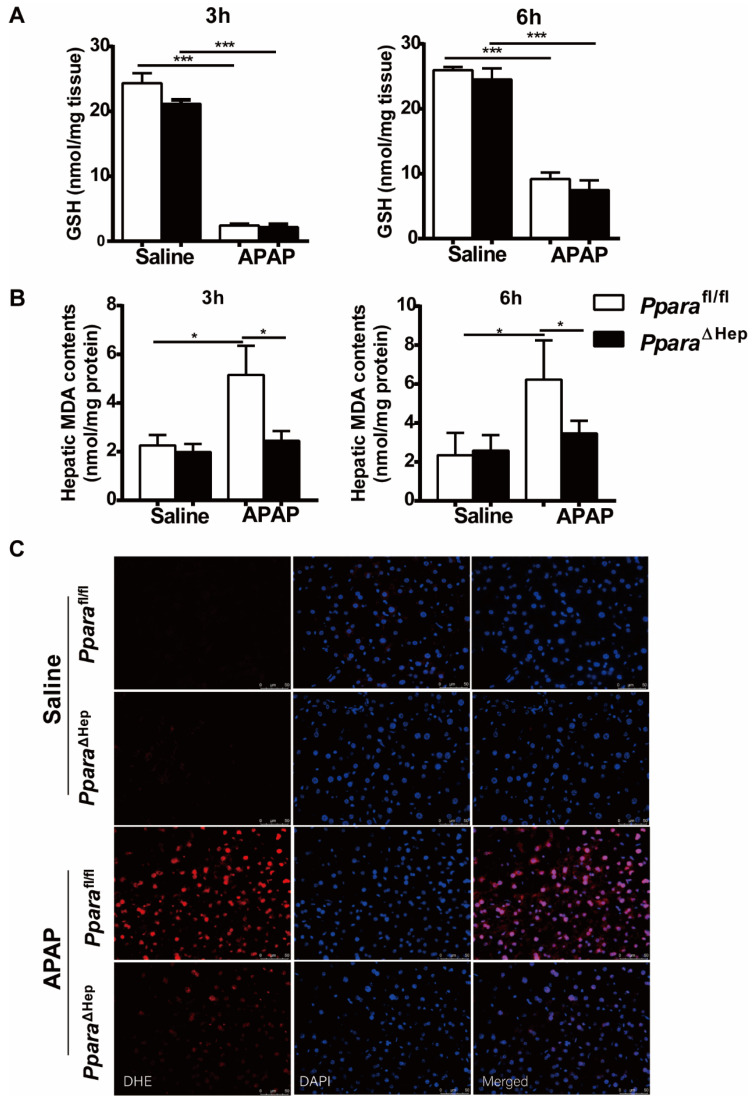
** Disruption of *Ppara* in hepatocytes decreases APAP-induced oxidative stress.** Eight- to ten-week-old male *Ppara*^ΔHep^ mice and* Ppara*^fl/fl^ mice were treated with APAP or saline. Liver tissues were collected at 3 hours and 6 hours after APAP treatment. **(A)** Hepatic GSH content were measured. **(B)** Malondialdehyde (MDA) contents were measured in the liver. **(C)** Representative images of dihydroethidium (DHE) staining (with DHE in red and DAPI in blue) in the liver. Data were represented as means ± SEM (n=5-8/group). *P<0.05, ***P<0.001.

Furthermore, hepatic levels of GSH were almost completely depleted and comparable at 3 h and 6 h after APAP injection in both groups of mice (Fig. 4A). This was consistent with previous studies showing that the liver content of GSH was exhausted after APAP treatment 19-21. These results suggested that the associated protective role of *Ppara*^ΔHep^ mice in APAP-induced liver injury was not due to APAP bioactivation and detoxification.

### Disruption of PPARα in hepatocytes decreases APAP-induced oxidative stress

Previous studies revealed that hepatic oxidative stress was the key mechanism in APAP hepatotoxicity 5, 6 and that up-regulation of PPARα activation by Wy14,643 inhibited fatty acid oxidation after APAP treatment, resulting in suppression of lipid peroxidation and the production of superoxide, the major component of reactive oxygen species (ROS) 16, 22. To further explore the mechanisms by which *Ppara*^ΔHep^ mice protected against APAP-induced liver injury, the hepatic content of malondialdehyde (MDA, lipid peroxidation markers) and dihydroethidium (DHE) staining for superoxide in liver tissue were examined. As illustrated in Fig. 4B, the hepatic level of MDA was lower in the APAP-treated *Ppara*^ΔHep^ mice than in the *Ppara*^fl/fl^ mice. Notably, DHE staining further confirmed that *Ppara*^ΔHep^ mice greatly diminished ROS production after APAP treatment compared to that of *Ppara*^fl/fl^ mice 3 hours post-APAP injection (Fig. 4C). These findings suggested that the protection of *Ppara*^ΔHep^ mice in APAP-induced liver injury relied on amelioration of APAP-induced oxidative stress.

### Disruption of PPARα in hepatocytes increases the IL-6/STAT3 signaling pathway after APAP treatment

**Figure 5:**
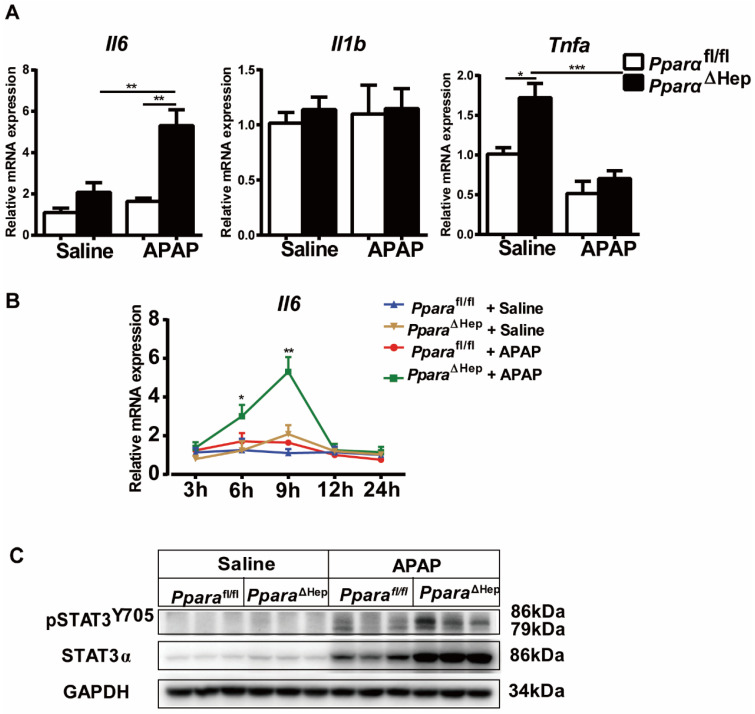
** Hepatocyte PPARα deficiency increases the IL-6/STAT3 signaling pathway after APAP treatment.** Eight- to ten-week-old male *Ppara*^ΔHep^ mice and* Ppara*^fl/fl^ mice were treated with APAP or saline. Liver tissues were collected 9 hours after APAP treatment. **(A, B)** Inflammatory cytokines were determined by qRT-PCR. **(C)** Hepatic protein levels of STAT3 and phosphorylation of STAT3 determined by Western blot. Data are presented as the means ± SEM (n=5-7/group), *P<0.05, ***P<0.001.

PPARα is known to regulate anti-inflammatory effects by trans-repression mechanisms, frequently targeting NF-kB and AP-1 signaling 23. We next investigated inflammatory cytokines in the liver. As illustrated in Fig. 5, qRT-PCR indicated that the gene expression of the inflammatory cytokine *Il6* was elevated over time and peaked at 9 hours in *Ppara*^ΔHep^ mice compared to *Ppara*^fl/fl^ mice after APAP treatment (Fig. 5A, B), and the mRNA levels of *Tnfa* and *Il1b* were unchanged (Fig. 5A). Activation of the IL-6/STAT3 pathway plays an important role in promoting liver regeneration after APAP treatment 24, and hepatocyte-specific STAT3 knockout inhibits hepatocyte proliferation and liver regeneration, aggravating APAP-induced liver injury 25. We next detected the levels of STAT3 phosphorylation at 9 hours after APAP treatment. As shown in Fig. 5C, STAT3 phosphorylation was upregulated in *Ppara*^ΔHep^ mice compared to *Ppara*^fl/fl^ mice after APAP treatment, indicating that STAT3 may be involved in the protection of APAP-induced liver injury in *Ppara*^ΔHep^ mice.

## Discussion

APAP-induced liver injury is a major public health problem that is strongly linked with acute liver failure. Most cases come from an unconscious ingestion of cold medicine containing APAP 1, 2. The majority of APAP is detoxified by the phase II enzymes UGT and SULT, while a small part is converted by P450 enzymes to the reactive metabolite NAPQI 3, 4. At therapeutic doses, the formation of NAPQI was detoxified by GSTM enzymes or directly bound to GSH to yield a GSH conjugate. After taking toxic doses of APAP, however, GSH is exhausted by conjugation. The highly reactive intermediate metabolite NAPQI accumulates, resulting in acute hepatic necrosis 4. Thus, it is essential to control the formation of toxic metabolites produced by APAP metabolism, which is known to be regulated by nuclear receptors 9. PPARα is a member of the nuclear receptor superfamily. Whole-body *Ppara^-/-^* mice show impaired fatty acid oxidation and accumulation of liver and serum levels of acylcarnitines, resulting in deterioration of APAP hepatotoxicity 15. However, PPARα is also expressed in other organs to function in fatty acid β-oxidation 10. Whether the role of PPARα in hepatocytes in APAP-induced liver injury is still unknown. Here, we explored the impact of hepatocyte-specific deletion of PPARα on APAP-induced liver injury *in vivo*. The present study demonstrates that hepatocyte-specific disruption of PPARα plays an important protective role against APAP-induced liver injury by reducing APAP-induced oxidative stress and upregulating the IL-6/STAT3 signaling pathway to promote liver repair.

Although it is not clear whether drugs exert hepatotoxic effects, PPARα may be involved in the hepatotoxic mechanism. In this paper, we found that the expression of PPARα was significantly decreased in DILI patients compared to HCs. This result suggested that PPRAα plays a major role in the process of drug-induced liver injury. APAP bioactivation was the foremost process associated with APAP-induced liver injury. Our data revealed that the protective effect of *Ppara*^ΔHep^ against APAP-induced liver injury was not due to APAP bioactivation and detoxification. This supports the concept that PPARα does not affect APAP metabolism 26. Additionally, we found that GSH was exhausting, which agreed with a previous study showing that GSH was exhausting following APAP overdosing 27. However, no differences in hepatic GSH content after APAP treatment were found between *Ppara*^ΔHep^ mice and *Ppara*^fl/fl^ mice. This result suggested that the protection of *Ppara*^ΔHep^ mice against APAP toxicity is not due to APAP metabolism.

Hepatic oxidative stress triggered by inhibition of mitochondrial respiration through the covalent binding of NAPQI to mitochondrial proteins is a central element of APAP-induced liver injury 5, 6. Excessive accumulation of ROS induces mitochondrial permeability transition (MPT) and the cessation of ATP generation, ultimately leading to hepatocyte necrosis 5. Our present study revealed that APAP induced significantly elevated ROS production in *Ppara*^fl/fl^ mice but diminished ROS production in almost all *Ppara*^ΔHep^ mice, as evidenced by the markedly lower levels of hepatic MDA content and decreased number of DHE-positive cells. Thus, hepatocyte-specific PPARα deficiency protects against APAP-induced liver injury by suppressing liver oxidative stress. However, previous studies revealed that the PPARα activators fenofibrate and Wy-14643 blocked APAP-induced oxidative stress, while whole-body *Ppara^-/-^* mice did not 15, 16. The induction of the PPARα target gene UCP2 might be associated with a protective role, as confirmed by the sensitivity of *Ucp2^-/-^* mice to APAP-induced toxicity 16.

It is well-known that PPARα signaling prevents the induction of mitochondrial and ER stress, and mitochondrial β-oxidation activity was significantly down-regulated in the livers of *Ppara*-null mice, PPARα agonist improves hyperglycemia-induced oxidative stress 28-31. There is some evidence that PPARα also has anti-inflammatory effects by counteracting nuclear factor kappa B (NF-κB) pathway 32.The results from the study of human primary hepatocytes treated with PPARα agonists also validate the anti-inflammatory role of PPARα 33. A recently study on autoimmune myocarditis reported PPARα may play an important role by regulating IL-6/STAT3 pathway on Th17 cell differentiation which contributes to inflammatory response 34. And more studies have also showed that PPARα agonists inhibits the expression of IL-6, while PPARα knockout mice displays an increase in IL-6 secretion when stimulated with LPS 35-38. Besides that, PPARα was proved anti-tumor effects in hepatoma cells by regulating NF-κB signaling 39. More importantly, macrophage-specific *Ppara* deletion did not impact Wy-14,643 induced up-regulation of lipid metabolism, cell proliferation, or DNA damage and repair-related gene expression. It has been proved that hepatomegaly and hepatocyte proliferation were observed in wild-type and *Ppara*^ΔMac^ mice, but not *Ppara*^ΔHep^ and whole-body *Ppara*-null mice. Furthermore, down-regulation of interleukin (IL)-15 and IL-18 after PPARα-agonist treatment was relied on the presence of macrophage PPARα. Growing evidence indicates PPARα may play the different roles in the different cell types, tissues, and underlying conditions 18, 40. So, the reasons may be that the effect of PPARα on APAP-induced liver injury is likely dependent on PPARα activity in different cells. Other cells, such as hepatic nonparenchymal cells, might play an important role in APAP-induced liver injury. This needs to be further investigated.

PPARα is known to interact with proinflammatory transcription factors, such as NF-κB, cJun and p65, to negatively regulate their target genes, *Il1b*, *Il6* and *Tnfa* 23. In this paper, we found that the mRNA levels of *Il6* were significantly increased in *Ppara*^ΔHep^ mice after APAP treatment, while *Il1b* and *Tnfa* were not. In the liver, STAT3, which is mainly activated by IL-6, IL-10 and IL-22, has been shown to play an important role in promoting liver proliferation and protecting against a variety of toxin-induced liver injuries 24, 41, 42. Furthermore, a previous study reported that activation of the IL-6/STAT3 pathway played an important role in promoting liver regeneration after APAP treatment 24, and hepatocyte-specific STAT3 knockout inhibited hepatocyte proliferation and liver regeneration, aggravating APAP-induced liver injury in the early phase 25. In this study, we revealed that APAP induced upregulation of STAT3 phosphorylation in *Ppara*^ΔHep^ mice compared to *Ppara*^fl/fl^ mice, indicating that STAT3 may be involved in promoting hepatocyte proliferation and liver regeneration in *Ppara*^ΔHep^ mice, which may be attributed to the protection of APAP-induced liver injury in *Ppara*^ΔHep^ mice compared to *Ppara*^fl/fl^ mice.

Altogether, the present study performed in *Ppara*^ΔHep^ mice after APAP overdose demonstrated that hepatocyte-specific PPARα deficiency plays an important protective role against APAP-induced oxidative stress and increases the IL-6/STAT3 signaling pathway to promote liver repair.

## Supplementary Material

Supplementary figures and table.

## Figures and Tables

**Table 1:** A list of mouse primer sequences for q-PCR

Genes	NCBI Refseq	Primers (5'-3')
*Cyp1a2*	NM_009993.3	F: GGAGCACTACCAAGACTTCAA
		R: TCAAAGCCAGCTCCAAAGATGT
*Cyp2e1*	NM_021282.2	F: TAGAAGTTGGAACCTGCCCC
		R: AGCGCTTTGCCAACTTGGTT
*Cyp3a11*	NM-007818.3	F: CATTGAGGAGGATCACACACAC
		R: GTCCCATATCGGTAGAGGAGCA
*Gstm1*	NM_010358.5	F: TCCGTGCAGACATTGTGGAG
		R: CTGCTTCTCAAAGTCAGGGTTG
*Gstm2*	NM_008183.3	F: GTTGGCCATGGTTTGCTACA
		R: CATAGGTGACCTTGTTCCCTG
*Ugt1a1*	NM_201645.2	F: TCAGCAATTGCCATAGCTTTC
		R: GTTGGTGGAATCAACTGCCT
*Sμlt2a1*	NM_001111296.2	F: CCAAGGCGATCTATCTCGTG
		R: ATAGAACATTTCCTTTGAGGAACC
*Mrp3*	NM_029600.3	F: GGACCATGAAGCCTTGCAAAATG
		R: CTCTCATGAACTGCTTGCGG
*Ppara*	NM_001113418	F: CCCTGAACATCGAGTGCGAA
		R: TTCGCCGAAAGAAGCCCTTA
*Cyp4a10*	NM_010011.3	F: AAGGGTCAAACACCTCTGGA
		R: GATGGACGCTCTTTACCCAA
*Cyp4a14*	NM_007822.2	F: AGCAAACTGTTTCCCAATGC
		R: ACCCCTCTAGATTTGCACCA
*Acox1*	NM_001271898	F: CCTGATTCAGCAAGGTAGGG
		R: TCGCAGACCCTGAAGAAATC
*Fgf21*	NM_020013	F: CTCCAGCAGCAGTTCTCTGA
		R: CCTGGGTGTCAAAGCCTCTA
*Il6*	NM_031168	F: ACCAGAGGAAATTTTCAATAGGC
		R: TGATGCACTTGCAGAAAACA
*Il1b*	NM_008361.4	F: GGTCAAAGGTTTGGAAGCAG
		R: TGTGAAATGCCACCTTTTGA
*Tnfa*	NM_013693	F: AGGGTCTGGGCCATAGAACT
		R: CCACCACGCTCTTCTGTCTAC

## Abbreviations

APAP: acetaminophen
AILI: APAP-induced liver injury
PPARα: peroxisome proliferator-activated receptor α
SULT: sulfotransferase
UGT: UDP-glucuronosyltransferases
NAPQI: n-acetyl-*p*-benzoquinone-imine
GST: glutathione s-transferase
Mrp3: multidrug resistance-associated protein 3
CYP450: cytochrome p450
ROS: reactive oxygen species
DILI: drug-induced liver injury
HC: healthy control
ALT: alanine aminotransferase
AST: aspartate aminotransferase
GSH: glutathione
MDA: malondialdehyde
DHE: dihydroethidium
ICAM: intercellular adhesion molecule
