# Supplementary material for: Disruption of peroxisome proliferator-activated receptor α in hepatocytes protects against acetaminophen-induced liver injury by activating the IL-6/STAT3 pathway
Source: Int J Biol Sci. 2022 Mar 6;18(6):2317–28. doi: 10.7150/ijbs.69609 (PMC8990472; doi:10.7150/ijbs.69609)
Supplement: Supplementary file 1 — Supplementary figures and table. [file ijbsv18p2317s1.pdf]

Supplementary Figure 1. Hepatic expression of PPARα is decreased in DILI patients compared with HCs. Activation of PPARα in drug-induced liver injury (DILI) and healthy controls (HCs) is shown in Fig 1. (A) Representative images of human liver sections from patients with DILI and from HC subjects. Liver sections were immunostained with an anti-PPARα antibody. (B) PPARα^{+} cells were counted in 10 randomly selected high-power fields. Data were represented as means ± SEM (n=11/DILI, n=3/HC). *P<0.05.

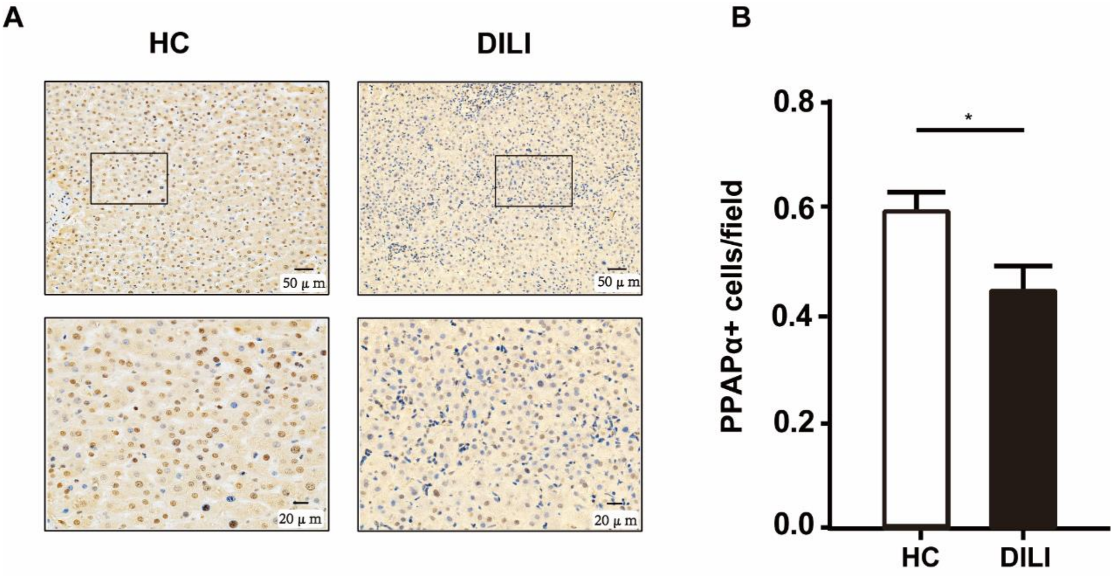

Supplementary Figure 2. The liver body weight ratio is unchanged in *Ppara*$^{fl/fl}$ and *Ppara*$^{\Delta Hep}$ mice after APAP injection. *Ppara*$^{fl/fl}$ and *Ppara*$^{\Delta Hep}$ mice were subjected to intraperitoneal injection with 400 mg/kg APAP dissolved in warm saline or an equal volume of saline. (A) Representative morphological view of liver from each group 9 hours after APAP treatment. (B) Liver body/weight ratio was measured. n=5-7/group.

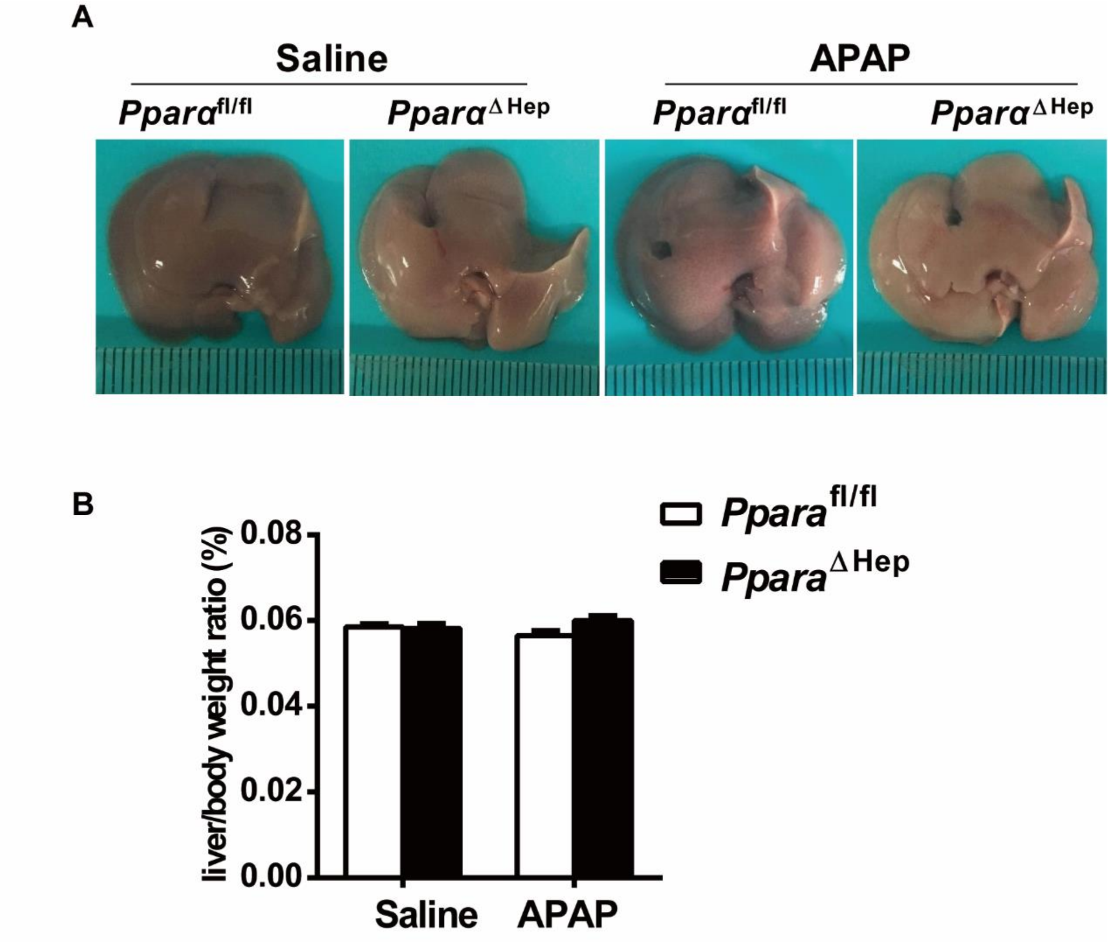

Supplementary Table 1. Clinical characteristics of DILI patients and healthy controls

No.	Gender	Age	ALT(IU/ml)	AST(IU/ml)	TBil(μmol/L)	DBil(μmol/L)	PT(s)
DILI Pt.1	F	24	219	374	87.6	73.7	13.3
DILI Pt.2	F	22	552	200	65	49	12.4
DILI Pt.3	F	57	397	223	20	17	11.2
DILI Pt.4	M	54	226	81	11.4	1.6	10.8
DILI Pt.5	F	55	165	180	164	70	13.1
DILI Pt.6	M	54	1943	439	61	43	14
DILI Pt.7	M	45	672	313	42	14.8	12.8
DILI Pt.8	F	34	240	130	23	11	11.1
DILI Pt.9	F	57	180	87	79	64	11.9
DILI Pt.10	M	70	550	247	51	32	12.6
DILI Pt.11	M	86	165	151	76	51	12.7
HC. 1	M	46	25	21	16	12	12
HC. 2	M	55	36	25	10	6	11
HC. 3	F	42	17	16	13	8	12.4
